# GOLPH3 and YB-1 Are Novel Markers Correlating With Poor Prognosis in Prostate Cancer

**DOI:** 10.14740/wjon952w

**Published:** 2015-12-31

**Authors:** Nehad M. R. Abd El-Maqsoud, Nisreen A. A. Osman, Amr M. A. El-Hamid, Tarek K. Fath El-Bab, Ehab M. Galal

**Affiliations:** a Faculty of Medicine, Minia University, Egypt

**Keywords:** GOLPH3, YB-1, Prostate cancer, Immunohistochemistry

## Abstract

**Background:**

Prostate cancer is a common and aggressive cancer among men. Despite advances in the treatment, the mechanisms involved in progression are still unclear. New prognostic markers should be explored for better design of patient-specific therapeutic regimens.

**Methods:**

This study was performed on 120 patients stratified as 76 with prostatic carcinoma, 12 with low-grade prostate intraepithelial lesion, 12 with high-grade prostate intraepithelial lesion and 20 with benign prostate hyperplasia. Immunohistochemical study was done for Golgi phosphoprotein 3 (GOLPH3) and Y-box binding protein-1 (YB-1) analysis. Correlation with clinicopathological data and overall survival was analyzed.

**Results:**

Both GOLPH3 and YB-1 showed increased expression from benign to malignant tumors. In prostatic carcinoma, cytoplasmic GOLPH3 was associated with Gleason score, stage and androgen receptor (P = 0.034, P < 0.001, and P = 0.008 respectively). Nuclear YB-1 expression was associated with Gleason score and androgen receptor (P = 0.018 and P = 0.024 respectively). Cytoplasmic YB-1 expression was associated with Gleason score, stage and androgen receptor (P = 0.008, P = 0.027, and P < 0.001 respectively). High Gleason score (P = 0.004), high stage (P < 0.001) and androgen receptor (P = 0.006) were the only detected adverse prognostic clinicopathological factors. Moderate/intense GOLPH3 and high nuclear and cytoplasmic YB-1 expression were correlated with shorter overall survival (P < 0.001, P = 0.020, and P < 0.001 respectively). In the multivariate analysis, moderate/intense GOLPH3 expression was the only predictor of overall survival (P = 0.025).

**Conclusions:**

High GOLPH3 and nuclear/cytoplasmic YB-1 expression correlated with poor prognosis in prostate cancer. Both markers can be promising targets for new treatment strategies.

## Introduction

Prostate cancer (PC) is the second most common malignancy among men in Europe and America [1]. In Egypt, it ranks the sixth (4.27%) and the incidence increases with aging [2]. PC is an aggressive disease and many patients die of metastatic cancer [3]. Despite advances in the treatment of PC, the mechanisms involved in progression and recurrence are still unclear. New prognostic markers should be explored for better design of patient-specific therapeutic regimens.

The specific diagnosis of prostatic intraepithelial neoplasia (PIN) has been a matter of debate considering its clinical significance and relationship to PC. PIN was simply classified into low-grade PIN (LGPIN) and high-grade PIN (HGPIN) [4]. LGPIN is associated with about 16% risk of developing PC; HGPIN carries about 21% risk that is close to the risk following the diagnosis of benign prostate hyperplasia (BPH) which is 20% [5].

The prostate gland development depends mainly on androgen through binding its androgen receptor (AR). Moreover, AR is important in the progression of PC being expressed in most of androgen-independent or hormone refractory PC. AR has a role in the deregulation of several oncogenes and tumor suppressor genes. So, its mutation may lead to the failure of endocrine therapy of PC [6].

Golgi phosphoprotein 3 (GOLPH3), also known as GPP34, GMx33, or MIDAS, is a membrane protein with a molecular weight of 34 kDa that plays a role in anterograde and retrograde Golgi trafficking and interactions with the cytoskeleton to maintain Golgi structure [7]. It is a promising marker in cell biology. Previous studies showed the presence of GOLPH3 as an oncogene encoded by a gene on chromosome 5p13 in several human cancers including breast, oral tongue, prostate, kidney and liver cancers [8-13]. In addition, the overexpression of GOLPH3 has been indicated to be correlated with clinically aggressive behavior of cancers and the prognosis of patients [13-15].

GOLPH3 promotes cell transformation and tumor growth by activating mammalian target of rapamycin (mTOR-YB-1) signaling pathway. Tumor cells overexpressing GOLPH3 are more sensitive to rapamycin [8, 15]. However, how GOLPH3 regulates cell migration and invasion is largely unknown.

Mammalian Y-box binding protein-1 (YB-1) is a member of the DNA/RNA-binding family of proteins that are involved in DNA repair, mRNA transcription, splicing, translation and stabilization [16]. YB-1 is related to cell proliferation, anti-apoptosis, and epithelial-mesenchymal transition as well as castration resistance in PC [17]. It is reported that YB-1 translocation from the cytoplasm to the nucleus stimulates transcription of a number of genes encoding proteins responsible for drug resistance, disease recurrence, metastasis and poor prognosis in various cancers [18, 19]. YB-1 acts as a biomarker for predicting the efficacy of high-dose chemotherapy in breast cancer [20]. Increases in YB-1 protein expression have also been described for other malignant tumors, such as osteosarcoma, PC, pancreatic adenocarcinoma, colorectal carcinoma, and glioblastoma, indicating the clinical impact of YB-1 for the progression of these malignant diseases [15, 18].

Little is known about correlation between GOLPH3 and YB-1 expression and prognosis in patients with PC. In the present study, we investigated the expression of GOLPH3, YB-1 in PC compared to its expression in BPH and PIN. Their correlation with clinicopathological features and their prognostic value for patients’ survival was also studied.

## Materials and Methods

### Patients’ data

This study was performed on 120 patients in the period extending from 2009 to 2014; these patients had undergone prostatectomy, transrectal prostate biopsy under ultrasound guidance, or transurethral resection of the prostate. Seventy-six (63.3%) patients suffered from PC, 12 (10%) cases were diagnosed with HGPIN and 12 (10%) with LGPIN. Twenty (16.7%) patients were diagnosed with BPH. All the specimens were fixed with 4% buffered formalin and embedded in paraffin. All the slides were blindly reviewed by two pathologists, and a final diagnosis was reached.

Clinical data, including Gleason score (GS), baseline prostate-specific antigen (PSA), stage and follow-up status, were retrospectively obtained from patients’ files at Minia University Hospital and Minia Oncology Center, Egypt. The data analysis was approved by our hospital review board.

### Immunohistochemical procedure

Slides were deparaffinized in xylene, rinsed with phosphate-buffered saline (PBS), and subjected to epitope retrieval treatment in 0.01 M citrate buffer solution (pH 6.0) for 2 min by a microwave. The sections were cooled at room temperature and further rinsed with PBS three times for 5 min each. The sections were then incubated with 0.3% hydrogen peroxide at room temperature for 10 min to quench the endogenous peroxidase activity and rinsed three more times for 5 min each. Sections were subsequently incubated with the anti-androgen receptor antibody (Abcam, rabbit polyclonal, 1:500), anti-GOLPH3 primary antibody (Abcam, ab113649; rabbit polyclonal; 1:100 dilutions) and anti-YB-1 antibody (Abcam, ab12148; rabbit polyclonal; 1:500), overnight at 4 °C. Then sections were washed with PBS and incubated with biotinylated secondary antibody for 30 min at room temperature. Streptavidin was applied for 30 min at room temperature. The color reaction was performed using diaminobenzidine (DAB) solution for 5 min. Finally, the slides were washed, counterstained with Mayer’s hematoxylin, dehydrated, and mounted. A section of PC, breast carcinoma and human duodenum were used as a positive control for AR, GOLPH3 and YB-1 proteins respectively.

### Immunohistochemical analysis

The immunohistochemical results were determined by two independent pathologists who were blinded to the clinicopathological data. Tissue sections were stained and AR nuclear expression was assessed by evaluating the proportion and intensity of positively stained carcinoma cells. A score was assigned to represent the estimated percentage of positively stained carcinoma cells, where 0, none; 1, 1%; 2, 1-10%; 3, 10-33%; 4, 33-67%; and 5, ≥ 67%. An intensity score was assigned to represent the average estimated stain intensity in positive carcinoma cells, where 0, none; 1, weak; 2, intermediate; and 3, strong. Proportion and intensity scores were added to obtain a total score ranging from 0 to 8. Immunohistochemistry results were classified according to the total scores with 0 - 4 classified as low expression and 5 - 8 as high expression [21].

GOLPH3 expression was determined according to the procedure previously described [11]. Staining for GOLPH3 was considered as positive when cytoplasmic staining was observed in more than 10% of definite cells. In cases of positive staining, the intensity of stain was recorded as either weak (1+), moderate (2+), or intense (3+). Finally, for immunohistochemistry, results were classified to negative/low expression and moderate/intense expression.

To assess nuclear/cytoplasmic YB-1 expression, YB-1 positive cells were counted. If the proportion of YB-1-positive cells was either less than or greater than 10%, then it was classified either as low or as high expression [21].

### Statistical analyses

Statistical analysis was performed using SPSS16.0 software. Chi-square test and Fischer’s exact test were used to investigate the significance of the relationship between GOLPH3, YB-1 and the individual variables. Pearson correlation was used to determine the correlation between each examined antibody and each histopathological entity. The relationship between GOLPH3, YB-1 expression and their clinical outcomes was estimated through both univariate and multivariate analyses. The overall survival (OS) curves were estimated using the Kaplan-Meier method, while the differences in the survival curves were compared using the log-rank test. A multivariate analysis was performed using Cox’s regression model. P values ≤ 0.05 were of statistical significance.

## Results

### Clinicopathological data of patients

The mean age of BPH cases was 65.70 ± 6.93 years with a median of 65 years (range 50 - 78 years). For the examined 24 PIN cases, we found the mean age was 66.79 ± 6.63 years and a median of 67.50 years (range 50 - 76 years). Twelve cases showed LGPIN and 12 cases HGPIN.

The mean age of PC was 67.68 ± 6.64 years with a median of 67.50 years (range 49 - 82 years). Baseline PSA was less than 10 in 13 cases (17.1%), between 10 and 20 in 39 cases (51.3%) and greater than 20 in 24 cases (31.6%). GSs were stratified into Gleason 2 - 6 in 17 cases (22.4%), Gleason 7 in 38 cases (50%) and Gleason 8 - 10 in 21 cases (27.8%). Pathological T (pT) stage was classified as pT2 in 40 cases (52.6%), pT3 in 27 cases (35.5%) and pT4 in nine cases (11.8%). Perineural invasion was found in 42 cases (55.3%) and absent in 34 cases (44.7%). Lymph node metastasis was absent in 61 cases (80.3%) and found in 15 cases (19.7%). Regarding the stage grouping of cases, five cases (6.6%) were stage I, 18 cases (23.7%) were stage IIA, 14 cases (18.4%) were stage IIB, 21 cases (27.6%) were stage III and 18 cases (23.7%) were stage IV (Table 1).

**Table 1:** Patients’ Clinicopathological Parameters in Patients With PC (N = 76)

Clinicopathological parameters	N	%
Age (years)		
≤ 60	8	10.5
> 60	68	89.5
Baseline PSA (ng/mL)		
< 10	13	17.1
10 - 20	39	51.3
> 20	24	31.6
Gleason score		
Gleason 2 - 6	17	22.4
Gleason 7	38	50
Gleason 8 - 10	21	27.8
pT status		
T2	40	52.6
T3	27	35.5
T4	9	11.8
Perineural invasion		
Absent	34	44.7
Present	42	55.3
pN status		
No	61	80.3
N1	15	19.7
Stage		
I	5	6.6
IIA	18	23.7
IIB	14	18.4
III	21	27.6
IV	18	23.7
Mortality		
Alive	30	39.5
Dead	46	60.5

pT status: pathological tumor stage; pN: lymph node status.

### GOLPH3 immunohistochemical cytoplasmic expression in the examined groups

**Figure 1:**
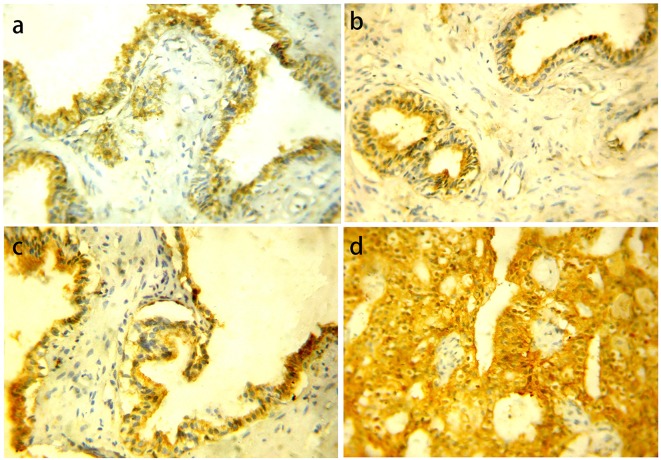
GOLPH3 protein cytoplasmic expression in different prostatic lesions using IHC. (a) GOLPH3 protein showed positive expression in BPH. (b) Positive GOLPH3 expression in LGPIN. (c) Positive GOLPH3 expression in HGPIN. (d) Positive GOLPH3 expression in PC. Original magnification, × 400 (DAB was used as the chromogen and hematoxylin as counterstain).

BPH showed negative/weak GOLPH3 expression in 15 (75%) cases and five (25%) cases showed moderate/intense expression. PIN cases were negative/weak GOLPH3 expression in 13 (54.2%) cases and 11 (45.8%) cases showed moderate/intense expression. LGPIN showed negative/weak GOLPH3 expression in eight (66.7%) cases and four (33.3%) cases showed moderate expression while HGPIN showed five (41.7%) negative/weak and seven (58.3%) moderate/intense expression. There were no statistically significant differences in GOLPH3 expression between LGPIN and HGPIN (P = 0.207). As regards carcinoma cases, we found negative/weak GOLPH3 expression in 24 (31.6%) cases and 52 (68.4%) cases showed moderate/intense expression (Fig. 1).

Statistically significant differences were present among GOLPH3 expression in the three previous groups collectively (P = 0.001) and between BPH vs. PC (P = 0.0031), PIN vs. carcinoma (P = 0.002), while there was no statistically significant difference between BPH vs. PIN (P = 0.098).

### YB-1 immunohistochemical expression

**Figure 2:**
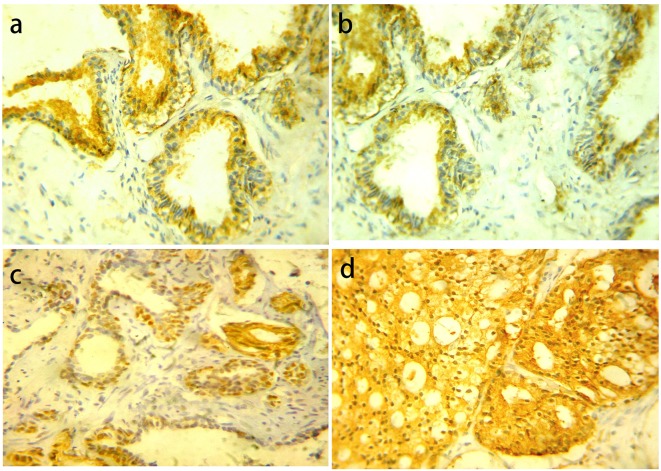
YB-1 protein expression in different prostatic lesions using IHC. (a) YB-1 protein showed cytoplasmic expression in BPH. (b) Positive cytoplasmic YB-1 expression in LGPIN. (c) Positive nuclear and cytoplasmic YB-1 expression in HGPIN. (d) Positive cytoplasmic and nuclear YB-1 expression in PC. Original magnification, × 400 (DAB was used as the chromogen and hematoxylin as counterstain).

YB-1 was distributed into both the nucleus and the cytoplasm. For nuclear YB-1 expression, all cases of BPH showed negative expression. PIN cases showed low YB-1 expression in 20 (83.3%) cases and high expression in four (16.7%) cases. LGPIN showed low YB-1 expression while all high expression cases were HGPIN. There was statistically significant difference in YB-1 expression between LGPIN and HGPIN (P = 0.047). Carcinoma cases showed low YB-1 expression in 48 cases (63.2%) and high expression in 28 cases (36.8%) (Fig. 2).

Statistically significant difference was present among the examined groups (P = 0.002). However, there was no statistically significant difference between PIN vs. PC (P = 0.363).

For cytoplasmic YB-1 expression, YB-1 expression showed high expression in six cases (30%) and low expression in 14 cases (70%) of BPH. PIN cases showed high YB-1 expression in 10 cases (41.7) while low expression was found in 14 cases (58.3%). There were no statistically significant differences in YB-1 expression between LGPIN and HGPIN (P = 0.660). In PC cases YB-1 showed high expression in 48 cases (63.2%) and low expression in 28 cases (36.8%).

YB-1 cytoplasmic expression was increased from BPH to PIN and from PIN to PC. Statistically significant differences were present among the examined groups (P = 0.013), between BPH vs. PIN (P = 0.011), PIN vs. PC (P = 0.004) while there was no statistically significant difference between BPH vs. PC (P = 0.077).

### Association of GOLPH3 and YB-1 immunohistochemical expression with clinicopathological parameters

Table 2 summarizes the immunohistochemical results of GOLPH3 expression in relation to different clinicopathological parameters in PC cases.

**Table 2:** Association of GOLPH3 Immunohistochemical Expression With Clinicopathological Parameters in Prostatic Carcinoma (N = 76)

Clinicopathological parameters	Intensity of GOLPH3 expression		P value
	Negative/low (%)	Moderate/intense (%)	
Age (years)			0.508
≤ 60	2 (25)	6 (75)	
> 60	22 (32.4)	46 (67.6)	
PSA (ng/mL)			0.160
< 10	7 (53.8)	6 (46.2)	
10 - 20	11 (28.2)	28 (71.8)	
> 20	6 (25)	18 (75)	
Gleason score			0.034
Gleason 2 - 6	6 (35.3)	11 (64.7)	
Gleason 7	16 (42.1)	22 (57.9)	
Gleason 8 - 10	2 (9.5)	19 (90.5)	
pT status			0.001
T2	20 (50)	20 (50)	
T3	3 (11.1)	24 (88.9)	
T4	1 (11.1)	8 (88.9)	
Perineural invasion			0.085
Absent	14 (41.2)	20 (58.8)	
Present	10 (23.8)	32 (76.2)	
pN status			0.017
No	23 (37.7)	38 (62.3)	
N1	1 (6.7)	14 (93.3)	
Stage			< 0.001
I	4 (80)	1 (20)	
IIA	12 (66.7)	6 (33.3)	
IIB	5 (35.7)	9 (64.3)	
III	2 (9.5)	19 (90.5)	
IV	1 (5.6)	17 (94.4)	
AR			0.008
Low expression	17 (45.9)	20 (54.1)	
High expression	7 (17.5)	32 (82.1)	
Mortality			< 0.001
Alive	22 (73.3)	8 (26.7)	
Dead	2 (8.3)	44 (84.6)	

Test of significance: Chi-squared and Fischer’s exact tests; P < 0.05 is considered significant. pT status: pathological tumor stage; pN: lymph node status; AR: androgen receptor.

Significant positive associations between GOLPH3 moderate/intense expression and Gleason score, pathological T (pT) stage, LNM (pN status) and stage were identified (P = 0.034, P = 0.001, P = 0.017 and P < 0.001 respectively). There was a positive association between GOLPH3 moderate/intense expression and AR (P = 0.008). No significant associations were seen in relation to patients’ age, PAS level and perineural invasion. YB-1 nuclear and cytoplasmic expression in relation to different clinicopathological parameters in PC cases was summarized in Table 3.

**Table 3:** Association of YB-1 Immunohistochemical Expression With Clinicopathological Parameters in Prostatic Carcinoma (N = 76)

Clinicopathological parameters	Nuclear YB-1 expression			Cytoplasmic YB-1 expression		
	Low (%)	High (%)	P value	Low (%)	High (%)	P value
Age (years)			0.0129			0.327
≤ 60	7 (87.7)	1 (12.5)		4 (50)	4 (50)	
> 60	41 (60.3)	17 (39.7)		24 (35.3)	44 (64.7)	
PSA (ng/mL)			0.731			0.237
< 10	9 (69.2)	4 (30.8)		7 (53.8)	8 (46.2)	
10 - 20	23 (59)	16 (41)		14 (35.9)	25 (64.1)	
> 20	16 (66.7)	8 (33.3)		7 (29.2)	17 (70.8)	
Gleason score			0.018			0.008
Gleason 2 - 6	13 (76.5)	4 (23.5)		7 (41.2)	10 (58.8)	
Gleason 7	27 (71.1)	11 (28.9)		19 (50)	19 (50)	
Gleason 8 - 10	8 (38.1)	13 (61.9)		2 (9.5)	19 (90.5)	
pT status			0.428			0.146
T2	28 (70)	12 (30)		18 (45)	22 (55)	
T3	15 (55.6)	12 (44.4)		9 (33.3)	18 (66.7)	
T4	5 (55.6)	4 (44.4)		1 (11.1)	8 (88.9)	
Perineural invasion			0.073			0.173
Absent	25 (73.5)	9 (26.5)		15 (44.1)	19 (55.9)	
Present	23 (54.8)	19 (45.2)		13 (31)	25 (69)	
pN status			0.277			0.031
No	40 (65.6)	21 (34.4)		26 (42.6)	35 (57.4)	
N1	8 (53.3)	7 (46.7)		2 (13.3)	13 (86.7)	
Stage			0.136			0.027
I	5 (100)	0 (0)		3 (60)	2 (40)	
IIA	13 (72.2)	5 (27.8)		11 (61.1)	7 (38.9)	
IIB	10 (71.4)	4 (28.6)		5 (35.7)	9 (64.3)	
III	12 (57.1)	9 (42.9)		7 (33.3)	14 (66.7)	
IV	8 (44.4)	10 (55.6)		2 (11.1)	16 (88.9)	
AR			0.002			< 0.001
Low expression	30 (81.1)	7 (18.9)		22 (59.5)	15 (40.5)	
High expression	18 (46.2)	21 (53.8)		6 (15.4)	33 (84.6)	
Mortality			0.107			< 0.001
Alive	22 (73.3)	8 (26.7)		20 (66.7)	10 (33.3)	
Dead	20 (56.5)	20 (43.5)		8 (17.4)	38 (82.6)	

Test of significance: Chi-squared and Fischer’s exact tests. P < 0.05 is considered significant. pT status: pathological tumor stage; pN: lymph node status; AR: androgen receptor.

There were positive associations between nuclear YB-1 expression and Gleason score and AR (P = 0.018 and P = 0.024 respectively). No significant associations were seen with other clinicopathological parameters. Cytoplasmic YB-1 expression was associated with Gleason score, LNM (pN status), stage and AR (P = 0.008, P = 0.031, P = 0.027 and P < 0.001 respectively). No significant associations were seen with other clinicopathological parameters.

### Correlations between immunohistochemical markers in different examined lesions

Correlations between immunohistochemical markers in different examined lesions are shown in Table 4.

**Table 4:** Correlations Between Immunohistochemical Markers in Different Lesions

Lesion	Marker expression		GOLPH3	Nuclear YB-1	Cytoplasmic YB-1
BPH	GOLPH3	Pearson correlation	-	0.103	0.649*
		Sig. (two-tailed)	-	0.665	0.002
	Nuclear YB-1	Pearson correlation	0.103	-	0.116
		Sig. (two-tailed)	0.665	-	0.627
	Cytoplasmic YB-1	Pearson correlation	0.649*	0.116	-
		Sig. (two-tailed)	0.002	0.627	-
PIN	GOLPH3	Pearson correlation	-	0.922**	0.399
		Sig. (two-tailed)	-	< 0.001	0.101
	Nuclear YB-1	Pearson correlation	0.922**	-	0.559*
		Sig. (two-tailed)	< 0.001	-	0.016
	Cytoplasmic YB-1	Pearson correlation	0.399	0.559*	-
		Sig. (two-tailed)	0.101	0.016	-
Carcinoma	GOLPH3	Pearson correlation	-	0.777**	0.438**
		Sig. (two-tailed)	-	< 0.001	< 0.001
	Nuclear YB-1	Pearson correlation	0.777**	-	0.611**
		Sig. (two-tailed)	< 0.001	-	< 0.001
	Cytoplasmic YB-1	Pearson correlation	0.438**	0.611**	-
		Sig. (two-tailed)	< 0.001	< 0.001	-

Test of significance: Pearson correlation. **Correlation is significant at the 0.01 level (two-tailed). *Correlation is significant at the 0.05 level (two-tailed).

In BPH cases, a significant positive correlation was noted between GOLPH3/cytoplasmic YB-1 expression (r = 0.649, P = 0.002). No significant correlation was noted between GOLPH3/nuclear YB-1 expression (r = 0.103, P = 0.665) and between nuclear/cytoplasmic YB-1 expression (r = 0.116, P = 0.627).

In PIN cases, a significant positive correlation was noted between GOLPH3/nuclear YB-1 expression (r = 0.922, P < 0.001) and between nuclear/cytoplasmic YB-1 expression (r = 0.559, P = 0.016) while no significant correlation was noted between GOLPH3/cytoplasmic YB-1 expression (r = 0.399, P = 0.101).

In PC cases, a significant positive correlation was noted between GOLPH3/nuclear and cytoplasmic YB-1 expression (r = 0.777, P < 0.001 and r = 0.438, P < 0.001 respectively) and between nuclear/cytoplasmic YB-1 expression (r = 0.611, P < 0.001).

### Prognostic value and survival analysis

The time of OS ranged from 4 to 60 months with a mean ± standard deviation (SD) of 26.20 ± 15.634 months and a median survival time of 21 months. The OS was 39.5%.

**Figure 3:**
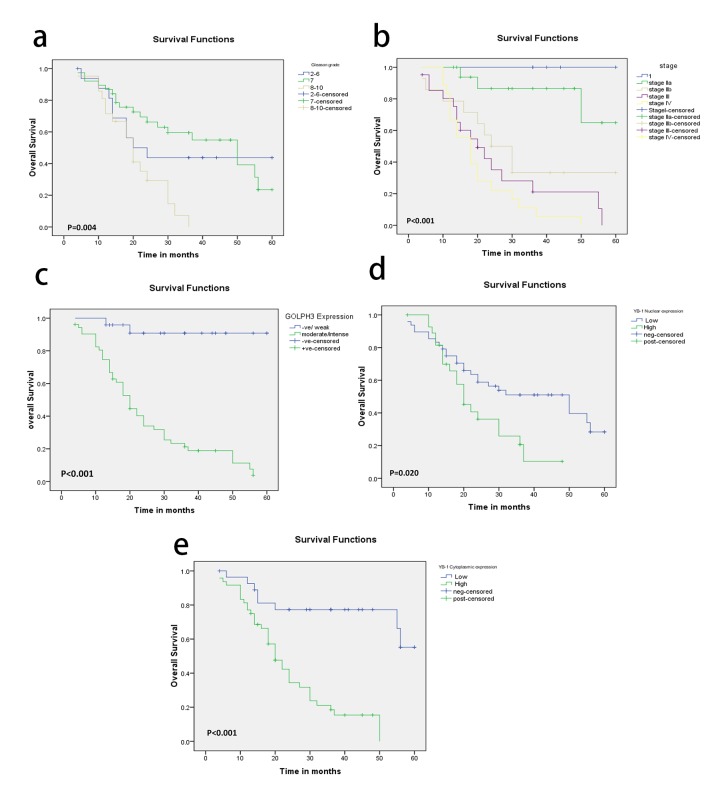
(a-e) Kaplan-Meier plots for overall survival in PC patients according to Gleason grade (a) and tumor stage (b), GOLPH3 expression (c), nuclear YB-1 (d) and cytoplasmic YB-1 expression (e) with significant P values using log-rank test.

High Gleason score (P = 0.004), pT status (P < 0.001), pN status (P < 0.001), high stage (P < 0.001) and AR (P = 0.006) were the only adverse prognostic clinicopathological factors. While OS rate was not significantly influenced by patients’ age (P = 0.896), PSA (P = 0.128), and perineural invasion (P = 0.064). Compared to patients with moderate/intense GOLPH3 expression, the patients whose tumor cells showed weak expression of GOLPH3 had significantly better outcomes in OS (P < 0.001). As regards nuclear and cytoplasmic YB-1 expression, we found that high YB-1 expression was associated with shorter OS and weak expression associated with better OS (P = 0.020 and P < 0.001 respectively) (Fig. 3).

In the multivariate analysis, moderate/intense GOLPH3 expression continued to be a significant predictor of OS (P = 0.025, Table 5).

**Table 5:** Multivariate Analyses of Overall Survival in Prostate Carcinoma Patients

Variables	HR	95% CI	P value
Grade	0.840	0.518 - 1.361	0.478
pT status	0.923	0.507 - 1.679	0.792
pN status	0.997	0.359 - 2.515	0.994
Stage	1.574	0.939 - 2.637	0.085
AR	1.086	0.603 - 2.451	0.585
GOLPH3	5.970	1.252 - 28.459	0.025
Nuclear YB-1	1.216	0.603 - 2.451	0.585
Cytoplasmic YB-1	2.100	0.731 - 6.036	0.168

Test of significance: Cox regression analysis. P < 0.05 is considered significant. HR: hazard ratio; CI: confidence interval.

## Discussion

PC is a serious disease that represents different challenges to the urologists, pathologists and oncologists [22]. Treatment of PC includes either surgical castration or androgen ablation therapy with transition from hormone refractory stage to metastatic stage during treatment [23]. The ordinary prognostic factors for PC include Gleason score, PSA and clinical stage. However, these factors seem to be inadequate to determine the possibility of recurrence or metastasis [24]. Therefore, reliable biomarkers have to be studied.

Recent studies have implicated GOLPH3 and YB-1 in integrin-mediated cell migration and invasion in glioma and breast cancer cells [9, 15, 25]. In glioma cells, the function of GOLPH3 requires mTOR and its effector YB-1. Upregulation of GOLPH3 is accompanied by increased activity of mTOR and YB-1. Both treatment with the mTOR inhibitor INK128 and YB-1 knockdown decrease the migration and invasiveness of glioma cells *in vitro* [15]. In non-invasive breast epithelial cells, YB-1 was shown to promote the epithelial-mesenchymal transition [26]. The invasion capacity of breast cancer cells depends on GOPLPH3 interaction with phosphatidylinositol 4-phosphate [27].

In the current study, moderate/intense cytoplasmic expression of GOLPH3 protein was found in 25% of BPH and in 68.4% of PC tissues and the difference was statistically significant. This result was similar to a previous study that reported 30% positivity in BPH and 64% in PC samples [28] but higher than previous studies that detected moderate/intense GOLPH3 expression in 5% of BPH, 8% of HGPIN and 37-40.66% of PC [8, 11]. Therefore, differences noted in the expression of GOLPH3 in PC compared with BPH and PIN may facilitate the clinical diagnoses of PC. Similar to a previous study in castration-resistant PC, GOLPH3 expression was positively correlated with Gleason score, tumor stage and lymph node metastasis [11]. On the contrary, no significant associations were found with Gleason score or tumor stage [28]. Collectively, these observations suggest that GOLPH3 acts as an oncogene in PC and may be a useful target for therapeutic interventions [11, 28, 29]. This result is supported by detection of GOLPH3 amplification in lung cancer [29]. Similarly, GOLPH3 has been considered as a novel oncogene involved in the development of cancer of the lung, ovary, breast, colon and prostate, as well as melanoma, rhabdomyosarcoma, and glioma [8, 30-32]. Emerging evidence has indicated that GOLPH3 was an androgen-regulated gene. In the present study, GOLPH3 expression was significantly associated with AR expression. This result was confirmed by another study that suggested that GOLPH3 upregulation may be involved in the progression from androgen-dependent to androgen-independent growth [28].

Previous studies in PC have shown that YB-1 played a role in enhancing cell proliferation, resistance to castration [21] and drug resistance [33]. Accordingly, therapy targeting YB-1 using adenovirus vector [34] and decoy peptide [35] can lead to suppression of PC proliferation.

In the present study, YB-1 was primarily expressed in the cytoplasm of benign prostate tissue at a low level and begins to increase with progression into malignant tissue. Following malignant transformation, YB-1 expression started to appear in the nuclei of both HGPIN and PC and this result was statistically significant despite of the little bit difficulty found during distinguishing nuclear from cytoplasmic staining. This finding was confirmed by other studies in prostate [36, 37]. Many studies have shown elevated YB-1 protein levels in malignant tissues compared with normal tissues, and higher levels of YB-1 have also been associated with higher tumor grade and poorer patient prognosis in different malignancies, including breast [38, 39], lung [40] and ovarian cancers [41]. Subsequently, YB-1 activities regarding oncogenic properties, cell proliferation and drug resistance are related to its nuclear translocation accompanied by altered gene transcription and chromosomal instability [21, 37].

Both high nuclear and cytoplasmic YB-1 expression was correlated with Gleason score as well as with AR expression in agreement with previous studies [33, 37-39]. On the contrary, high nuclear YB-1 expression was not correlated with Gleason score but correlated with advanced tumor stage [17]. In our study, only cytoplasmic expression was correlated with advanced stage. Many studies found that AR was a direct gene target of YB-1 and YB-1 was found to regulate AR transcription via binding to the AR promoter [21, 42]. In PC, YB-1 high expression converted androgen-dependent cells into castration-resistant cells. Thus, therapeutic targeting of YB-1 may be applicable in cases with AR splice variants in castration-resistant PC [21].

A significant positive correlation was noted between GOLPH3/nuclear and cytoplasmic YB-1 expression in PC cases of the present study. This finding was also reported in glioma cancer cells which demonstrated that overexpression of GOLPH3 increased the protein level of YB-1 and GOLPH3 up-regulation is accompanied by an increase of YB-1 level and mTOR activity, suggesting that GOLPH3 is located at the upstream of YB-1. The effect of GOLPH3 on enhancing migration and invasion of glioma cells could be abolished by either treatment with the mTOR inhibitor INK128 or by YB-1 knockdown [15].

In the current study like a previous study, high Gleason grade, advanced tumor stage, and AR were associated with shorter OS [11]. GOLPH3 positive expression was also associated with shorter OS. Therefore, the level of GOLPH3 expression in radical prostatectomy samples may be useful for predicting OS and can be an important parameter for the prognosis of PC patients. Previous studies have indicated that high level of GOLPH3 expression promotes tumorigenesis and progression of several types of malignancies including PC and was associated with poor prognosis [10, 14, 15, 25, 43]. Similarly, high nuclear and cytoplasmic YB-1 expression was associated with shorter OS. So, YB-1 overexpression can be regarded as a sign of poor prognosis and can be used as biomarker for tumor aggressiveness as reported in previous studies on PC, breast cancer, ovarian cancer and lung cancer [39-41, 44]. Both GOLPH3 and YB-1 enhance cell mortality. On the other hand, multivariate analysis indicated that GOLPH3 was the only significant prognostic factor of OS in all PC patients and this finding was also reported by another study [11]. On the contrary, neither nuclear nor cytoplasmic YB-1 expression was not associated with OS and it can be considered as independent prognostic factor. Therefore, GOLPH3 positive expression can be superior over YB-1 high expression as a poor prognostic marker for PC patients.

In conclusion, GOLPH3, nuclear/cytoplasmic YB-1 expression correlated with the Gleason score, AR expression and poor OS in PC tissues. Both markers work in the same molecular pathway and function as oncogenes. Based on these findings, we revealed that both GOLPH3 and YB-1 may be promising molecular targets for new therapeutic cancer strategies during the treatment of both androgen-dependent PC and castration-resistant PC.

### Clinical practice points

PC is an aggressive disease. Despite advances in the treatment, the mechanisms involved in progression and recurrence are still unclear. New prognostic markers should be explored for better design of therapeutic regimens. The role of GOLPH3 and YB-1 is still under research in PC. Immunohistochemical analysis of GOLPH3 and YB-1 in different lesions of the prostate that included 20 benign prostatic hyperplasia (BPH), 20 prostatic intraepithelial neoplasia (PIN) and 67 PC cases was done.

Moderate/intense cytoplasmic expression GOLPH3 expression was found in 25% of BPH cases, 45.8% of PIN cases and in 68.4% of PC cases. Regarding nuclear YB-1, all cases of BPH showed negative expression. High expression was detected in 16.7% of PIN cases and in 36.8% of PC cases. For cytoplasmic YB-1 expression, high expression was found in 30% of BPH cases, 41.7% of PIN cases and in 63.2% of PC cases.

Significant positive associations between GOLPH3 expression, YB-1, Gleason score, stage and AR were found. In PC cases, a significant positive correlation was noted between GOLPH3/nuclear and cytoplasmic YB-1 expression.

High Gleason score, high stage, AR, high GOLPH3, and high nuclear/cytoplasmic YB-1 expression were the only adverse prognostic factors. In the multivariate analysis, GOLPH3 expression continued to be a significant predictor of OS. Both markers can be promising targets for new treatment strategies.
